# Uncovering the Connection: PTSD and Road Accidents

**DOI:** 10.1192/j.eurpsy.2024.1381

**Published:** 2024-08-27

**Authors:** S. Bellafqih, Y. El Fatmaoui, S. Belbachir, A. Ouanas

**Affiliations:** Hôpital Arrazi, CHU Ibn Sina, Salé, Morocco

## Abstract

**Introduction:**

Post-Traumatic Stress Disorder (PTSD) is a psychiatric disorder that can occur after a traumatic event. It results in mental suffering and physical complications that profoundly alter personal, social, and professional life.

One can develop PTSD after experiencing a frightening event, for example: rape, the death of a loved one, war veterans, or following a car accident. In Morocco, traffic accidents cause, on average, nearly 3,500 deaths and 12,000 serious injuries per year.

**Objectives:**

Our main purpose is to evaluate the incidence of post-traumatic stress disorder in patients who are victims of traffic accidents and to identify key risk factors in the general population.

**Methods:**

This is a descriptive cross-sectional study through a questionnaire shared on social networks including a socio-demographic description, a clinical description, and the “Peri-traumatic Distress Inventory (PDI)” Scale to evaluate the risk of developing PTSD.

**Results:**

This study is based on 48 participants with 82.8% of females and 17.2% of men. The average age was 27.6. Most of the participants lived in urban areas (93%), a majority had higher education (93.1%), and 41.4% of the candidates had a physical impact of the accident.

According to PDI scale, 65% showed PTSD and the average score was 20.3. A score of 15 and above indicates significant distress.

**Conclusions:**

Our results confirm the presence of PTSD in victims of accidents. We propose a clinical reflection on the possible improvement of the care of people suffering from PTSD following a public road accident.

**Disclosure of Interest:**

None Declared

